# Fostering Palliative Care Through Digital Intervention: A Platform for Adult Patients With Hematologic Malignancies

**DOI:** 10.3389/fdgth.2021.730722

**Published:** 2021-12-17

**Authors:** Lefteris Koumakis, Fatima Schera, Heather Parker, Panos Bonotis, Maria Chatzimina, Panagiotis Argyropaidas, Giorgos Zacharioudakis, Michael Schäfer, Christine Kakalou, Christina Karamanidou, Jana Didi, Eleni Kazantzaki, Lydia Scarfo, Kostas Marias, Pantelis Natsiavas

**Affiliations:** ^1^Institute of Computer Science, Foundation for Research and Technology–Hellas (FORTH), Heraklion, Greece; ^2^Institute for Biomedical Engineering, Sulzbach, Germany; ^3^Atlantis Healthcare, London, United Kingdom; ^4^Institute of Applied Biosciences, Centre for Research and Technology Hellas, Thessaloniki, Greece; ^5^Center for Molecular Medicine, Central European Institute of Technology, Masaryk University, Brno, Czechia; ^6^Department of Hematology, University Hospital of Heraklion, Heraklion, Greece; ^7^Universita Vita-Salute San Raffaele, Milan, Italy

**Keywords:** palliative care, health informatics, digital intervention, mHealth, cancer, ePROs

## Abstract

Patient-reported outcomes (PROs) are an emerging paradigm in clinical research and healthcare, aiming to capture the patient's self-assessed health status in order to gauge efficacy of treatment from their perspective. As these patient-generated health data provide insights into the effects of healthcare processes in real-life settings beyond the clinical setting, they can also be viewed as a resolution beyond what can be gleaned directly by the clinician. To this end, patients are identified as a key stakeholder of the healthcare decision making process, instead of passively following their doctor's guidance. As this joint decision-making process requires constant and high-quality communication between the patient and his/her healthcare providers, novel methodologies and tools have been proposed to promote richer and preemptive communication to facilitate earlier recognition of potential complications. To this end, as PROs can be used to quantify the patient impact (especially important for chronic conditions such as cancer), they can play a prominent role in providing patient-centric care. In this paper, we introduce the MyPal platform that aims to support adults suffering from hematologic malignancies, focusing on the technical design and highlighting the respective challenges. MyPal is a Horizon 2020 European project aiming to support palliative care for cancer patients via the electronic PROs (ePROs) paradigm, building upon modern eHealth technologies. To this end, MyPal project evaluate the proposed eHealth intervention via clinical studies and assess its potential impact on the provided palliative care. More specifically, MyPal platform provides specialized applications supporting the regular answering of well-defined and standardized questionnaires, spontaneous symptoms reporting, educational material provision, notifications etc. The presented platform has been validated by end-users and is currently in the phase of pilot testing in a clinical study to evaluate its feasibility and its potential impact on the quality of life of palliative care patients with hematologic malignancies.

## Introduction

Definition of palliative care has radically changed in the last years from a focus on end-stage cancer to include the trajectory of all life-limiting conditions. Currently, World Health Organization (WHO) defines palliative care as “*an approach that improves the quality of life of patients and their families facing the problems associated with life threatening illness, through the prevention and relief of suffering by means of early identification and impeccable assessment and treatment of pain and other problems, physical, psychological and spiritual*”(1). According to this definition, palliative care follows an interdisciplinary approach and encompasses the patient, the family and the community in its scope. That notwithstanding, roles and goals of palliative care remain both contested and poorly understood by healthcare professionals, and more importantly, by patients and the public.

To this end, the aim of palliative care is to assess and aid the patient needs regardless of the place where they are cared for (e.g., at home, in a hospital, in a hospice care). Though frequently interpreted as end-of-life care, palliative care focuses on life and takes into consideration dying as a normal process, without any intention of hastening or postponing death. Its main goal is to preserve the best possible quality of life until death by supporting the patient not only through physical problems but other additional problems of a social, psychological, and spiritual nature. To this aim, palliative care is focusing not only on the patients themselves but also on their families. As the main purpose of palliative care is to improve quality of life, it can be offered to patients (and their families) who are receiving active treatment either in a curative fashion or aimed at disease control. In this modern view, the adoption of palliative care should not be restricted only to end-of-life or hospice care. It is an essential component of the trajectory of any serious chronic illness and a necessary component of care for patients with chronic diseases. Along this line, palliative care should begin at diagnosis and increases in “dosage” throughout the continuum of illness. Randomized controlled trials conducted in the US support the benefits of early integration of palliative care into clinical care. Though these results were not replicated in European studies, they deserve further investigation considering their potential relevance for the management of the patients with chronic conditions and in particular those diagnosed with solid tumors or hematological malignancies.

As palliative care is a highly personalized treatment paradigm, the patient is (or at least should be) an active member of the decision-making team. Thus, the communication between the patient and the respective healthcare team, is identified as a top-class priority, and eHealth tools could provide the means to move toward better communication and cooperation in terms of treatment decision making process.

Patient-reported outcomes (PROs) are an emerging paradigm of patient management and patient-doctor communication focusing on the patient reporting his/her health status instead of the doctor evaluating it. In the last 10 years, a number of digital interventions aiming to support palliative cancer care based on the electronic PRO (ePRO) systems have been evaluated. In (2), 24 relevant publications reviewed, concluding that the proposed digital health interventions are positively evaluated by end-users and that ePRO interventions could have a significant positive impact on health outcomes. Similarly, a recent meta-review presented by Finucane et al. (3) concluded that digital health interventions in palliative care could positively affect patients, especially regarding education, information sharing, decision-making, communication, and costs. More specifically, a number of research works highlight the benefits in specific aspects like physical activity (4), reduction in anxiety and drowsiness (5), fatigue, nausea, and insomnia (6). However, it has also been identified that palliative care digital interventions tend to be associated with advanced cancer stages, which suggests insufficient digital health research embracing the current definition of palliative care which does not only focus on the need to support end-of-life treatment (2).

This paper provides details for the design and development of the MyPal infrastructure, based on a user centered development process. More specifically, for the realization of MyPal, web and mobile applications have been developed including the MyPal mobile app, the healthcare professional tool and the administrator panel offering a unique user experience to patients, doctors/researchers and caregivers. The design and the implementation of the MyPal intervention is based on extending and adapting recent achievements from other projects, such as iManageCancer (7, 8), and major involvement of end-users. The key driver of the MyPal concept is “use case” scenarios that have been developed in an iterative fashion with the participation of all stakeholders (patients, family members, clinical care providers, and patient organizations) along with special attention to technology acceptance and potential barriers such as digital literacy.

The rest of the manuscript is structured as follows: Section Methods presents the methods used for the design, development and validation. In section Proposed digital intervention model we elaborate on the proposed digital intervention model and section Validation presents the results of the internal validation for the platform. In section Discussion we discuss the results, while section Conclusions concludes this paper and provides directions for future work.

## Methods

### Design Phase

An agile user-centered or “participatory design” methodology has been adopted to identify end-user needs and to design the overall MyPal platform. More specifically, focus groups, i.e., focused discussions among groups of end-user representatives, were conducted to elaborate on the use cases and identify potential restrictions and end-user requirements. To facilitate the discussion, a number of vignettes were designed, i.e., scenarios that present at an abstract level the envisioned functionalities of the platform to be developed with the help of one or more imaginary users of the platform. A vignette has been designed following an imaginary middle-aged male diagnosed with chronic lymphocytic leukemia (CLL) or myelodysplastic syndrome (MDS) in his journey. The vignette has been translated to the following languages: English, Greek, Italian, and Czech as focus groups were conducted in Greece, Italy and Czech Republic. A discussion guide has also been designed, consisting of a general comment section (addressing the platform as a whole) and several feature/functionality specific sections. The audio material of the focus groups was analyzed, and the main findings of each focus group were expressed as three separate lists of themes, namely positive comments, negative comments, and recommendations. The identified themes were additionally tagged according to the functionality they refer to and they were taken into account to guide the design of the platform. Each use case was checked against the relevant focus group themes to ensure that the resulting use case was compatible with the opinion of the corresponding end-users; in case of discrepancies, the use case was revised accordingly.

The patients engaged in this “participatory design process” were recruited via patient advocacy groups, approximately matching the targeted patients' profile. The enrolled patients' profile is outlined based on specific eligibility criteria defined by the respective clinical study protocol to be applied during the pilot phase of the project and can be summarized as follows: Adults (≥18 years); Diagnosed with CLL or small lymphocytic lymphoma (SLL), a condition equivalent to CLL, or MDS; Scheduled to receive any line of treatment for CLL/SLL or MDS or who have been previously exposed to any treatment for CLL/SLL or MDS; Able to understand and communicate in the respective language; Users of an Internet connected device (smartphone/tablet); life expectancy more than 3 months.

### Development Phase

The MyPal software architecture refers to the fundamental structures of the MyPal platform and the discipline of creating such structures and systems. The development phase followed a scrum methodology (9), which occurs in pieces of software and many iterations per piece. Such a methodology builds components in small steps taking into account the feedback and recommendations from end users or testers, enhances creativity and reassures that only what is needed will be built.

**Figure 1:**
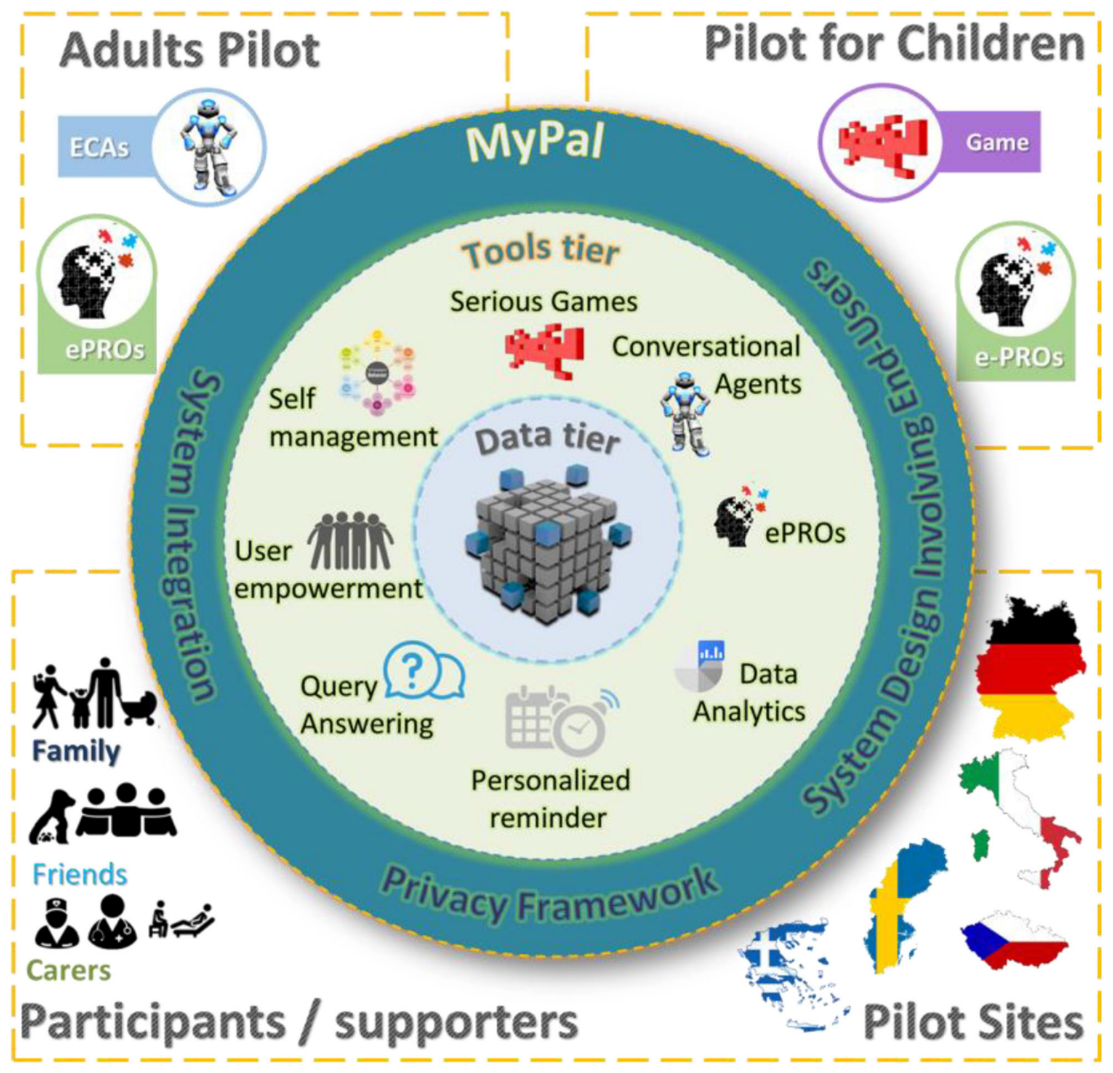
MyPal conceptual framework.

The conceptual framework of MyPal includes the integration of several building blocks supporting patients and their healthcare providers, having at its cornerstone advanced PRO systems, data reporting, storage and tools (Figure 1). The building blocks are organized in four tiers: (i) the **data tier**, which contains data extraction, transformation and serving functionalities, (ii) the **tools tier**, which contains the technology components, such as ePROs (via mobile/desktop apps and a game), self-management tools and psycho-emotional assessment, (iii) the **interaction tier**, which contains disruptive user interfaces for the end users, particularly adapted to their needs and preferences, and, finally, (iv) the **privacy and security tier** coping with user privacy and data security aspects.

The procedures of automatically generating, storing and exchanging personal data, in compliance with data security regulations, require a large number of functionalities offered by well-designed and interconnected building blocks. To this end, MyPal defines a modular architecture with interoperable functional blocks, combining, and going beyond the current state-of-the-art in ePROs with voice ePROs, interactive information access and intelligent recommendations of goals. All these technologies are combined in order to finally develop an innovative solution for an enhanced user interface, enabling patients, healthcare professionals (HCPs), and family caregivers to fully utilize their potential, knowledge, and valuable skill sets.

MyPal combines all the technological blocks to provide a unique user experience supported by electronic questionnaires, conversational agents and gamification to overcome the limitations in acceptability of traditional human-computer interaction techniques for the MyPal users.

A privacy and security layer wrap the whole architecture, data flow and interactions. The privacy-by-design approach (10), i.e., that data protection safeguards should be built into products and services from the earliest stage of development, has been addressed by the European Commission in the General Data Protection Regulation (GDPR). In MyPal, we adhere to the principles of “Privacy by Design,” i.e., a development method for privacy-friendly systems and services, thereby going beyond mere technical solutions, addressing organizational procedures, and business models as well.

### Validation Phase

The validation of the platform before being deployed in real-world conditions was identified as an important part of the overall participatory design process. It involved end-users, namely both patients and healthcare professionals via virtual and physical focus groups. More specifically, scenario-based “think-aloud” sessions were conducted to collect user feedback and identify potential issues before testing the platform in real-world settings. The walk-through usage scenario was divided into episodes, each describing a specific functionality/task pertaining to the MyPal platform (e.g., adding a new patient to the system). A “think aloud” approach was adopted during these meetings, where the participants were encouraged to be as talkative as possible while performing the requested actions, expressing their initial reactions, thoughts, worries and other considerations and also discouraging the validators from intervening or providing guidance. At the end of each meeting, the participant was asked to complete an anonymous short questionnaire either online (for the HCPs) or in printed forms (for the patients). The questionnaire included some general demographics questions, the questions of the Post-Study System Usability Questionnaire (PSSUQ) and 3 questions on general comments and recommendations for the MyPal platform.

More specifically, the virtual validation workshops with HCPs were conducted as one-on-one teleconference meetings. Each meeting lasted for ~1 h and was recorded for later analysis of end user behavior, comments, and reactions. Every participant received in advance an email containing a presentation of the walkthrough scenario, a consent form for the recording and a PDF file of a CLL-related scientific article to serve as a sample document to upload for the MyPal search functionality.

The validation focus groups with patients were organized by CERTH (Thessaloniki, Greece) engaging adult patients diagnosed with CLL, all living with the disease for an extended amount of time. This was deemed important for the validation purposes, since they had a good understanding of their disease and its management needs. It should be noted that these validation sessions focused on the MyPal mobile app as this was identified as the riskiest part of the specific intervention in terms of usability.

### Evaluation Phase

In order to evaluate the potential impact on both emotional and physical symptoms of the proposed platform on adult patients with hematological malignancies, a specialized clinical study has been designed aiming to investigate if it can lead to improved quality of life via a two-arms randomized control trial (RCT). To this end, a number of widely accepted measurement scales and standardized questionnaires are included, i.e., the EORTC QLQ-C30 General Questionnaire (11), the Euroqol EQ-5D (12), Integrated Palliative Care Outcome Scale –(IPOS) (13), Edmonton Symptom Assessment System (ESAS) (14), Brief Pain Inventory (BPI) (15) and Emotion Thermometer (ET) (16), as well the overall patient satisfaction with treatment is depicted via the European Organization for Research and Treatment of Cancer satisfaction with cancer care core questionnaire (EORTC PATSAT C33) (17). Moreover, other study specific parameters are also used as part of the intervention evaluation process, e.g., hospital visits, doctor visits, hospitalizations, medications, treatments, and investigations).

### Pilot for Adults

The study protocol was defined by MyPal-Adult Task Force which comprises of representatives of each involved clinical site as well as contributing members of the project's consortium. The participating clinical centers involved in the MyPal clinical trial are:

- Università Vita-Salute San Raffaele (USR)/IRCCS San Raffaele Hospital in Italy
- University General Hospital of Heraklion (PAGNI) in Greece
- General Hospital of Thessaloniki “George Papanikolaou” in Greece
- University Hospital Brno (FNBRNO) in Czech Republic
- Karolinska Institutet (KI) in Sweden

Participating center selection was based on disease-related established expertise for MDS and CLL, relevant experience in clinical trial design and conduct and longstanding collaboration in previous research projects. The MyPal4Adults study (ClinicalTrials.gov Identifier: NCT04370457) aims at improving the quality of life of patients with hematologic malignancies by implementing the use of palliative care at an early stage in the disease course. The study design is based on the comparison of a standard care arm with an experimental arm. Only patients randomized to the experimental arm will use the MyPal eHealth system and complete ePROs at pre-specified timepoints. Data collected will be analyzed to understand if MyPal intervention is leading to improved QoL and should be implemented as standard approach for patients with hematologic malignancies. The expected advantages will be analyzed in terms of both effectiveness and cost-effectiveness.

Prior to the recruitment, a rehearsal testing was performed, to evaluate the platform and assess its usability in clinical practice. Major technical issues were eliminated and feedback from participants was taken into consideration. As part of the clinical study protocol, initially, patients are informed, sign consent, and complete baseline questionnaires. Then each patient in the experimental “intervention” arm actively participates in the study regularly providing ePRO information for 6 months (main usage phase). After the first 6 months, there is a further 6 month “follow up” phase where only monthly questionnaire input is collected. Patients recruitment is currently ongoing at all clinical sites (BRN/UHB, PAGNI, USR, PAPANIKOLAU, and KI).

## Proposed Digital Intervention Model

### The MyPal Architecture

**Figure 2:**
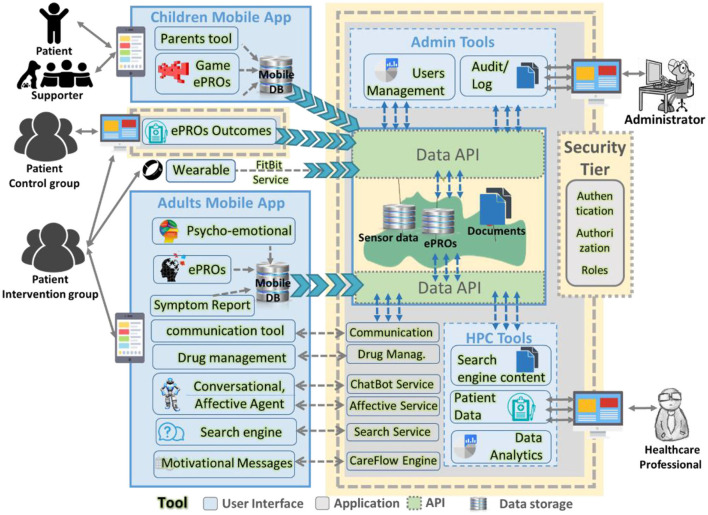
MyPal software architecture.

The architecture of the MyPal infrastructure, shown in Figure 2, includes various components and their interactions in the MyPal ecosystem. The architecture is a result of three software development cycles with constant interaction with end-users. It consists of eleven tools for the adult patient wrapped into one mobile application, seven tools for the healthcare professional served as a web application and tools for the administrator. MyPal also supports young patients (children and adolescents) and their careers in the CHILD study (18) with a serious game for patients, a mobile application for the carer and a web application for the healthcare professionals.

### MyPal Mobile Application

**Figure 3:**
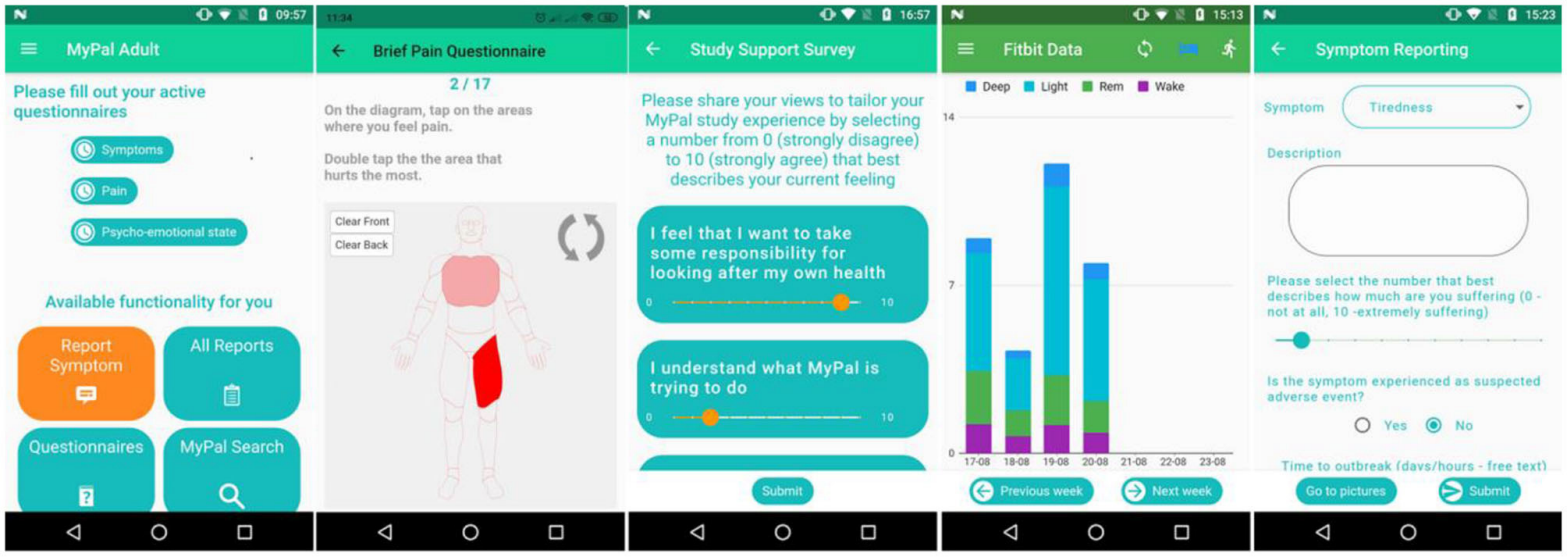
MyPal Mobile application screenshots. From left to right: (i) MyPal main menu, (ii) brief pain questionnaire, (iii) survey, (iv) activity data, (v) symptom reporting.

The MyPal mobile application was developed as a part of the MyPal platform for adults to advance palliative care for people with cancer by leveraging PRO systems through their customization to the personal needs of the cancer patient and their caregiver. In doing so, important variations in the patient's condition and quality of life (QoL) need to be identified as soon as possible. The app allows adults suffering from hematologic malignancies to capture and provide reports on QoL, side effects of cancer therapy, and medication adherence to their treating oncologists. The application provides users with functionalities for reporting questionnaire data, symptoms, medications, searching validated medical content and viewing the data collected from a wearable. Figure 3 shows some of the main tools from the MyPal mobile app while in the following we provide a short description of the available functionalities:

- The patient will be able to capture and send the following reports to the MyPal server:
- **Study Support survey** – a six-item questionnaire representing factors related to patient engagement (e.g., motivation, expectations, etc.). Depending on the answers to the questions, the patient will receive periodic motivational messages that address the factors in their survey responses in priority order. The patient will complete the survey three times during the study: at the start of the study, by the end of the third month, and the sixth month after the subject patient is enrolled in the MyPal Adult clinical trial.
- **Treatment Support survey** – a six-item questionnaire representing risk factors for non-adherence. Patients who are on treatment for CLL or MDS will be prompted to complete this via the app. The answers to the questions will guide the treating physician to conduct a tailored patient interview during the next appointment, using the HCP Discussion Tool in the MyPal platform. The patient will complete the survey three times during the study: at baseline, by the end of the third month, and the sixth month after the subject patient is enrolled in the MyPal Adult clinical trial.
- **Three questionnaires to capture symptoms, pain, and emotional state** to be completed weekly. These questionnaires represent corresponding standardized questionnaires: the Edmonton Symptom Assessment System (ESAS), Brief Pain Inventory (BPI) severity and Emotion Thermometers (ET).
- The patient will be directed to the MyPal web application to complete four additional **QoL** questionnaires on a monthly basis.
- The patient is notified when to complete a questionnaire.
- The patient can record and communicate his/her **symptoms** on an *ad hoc* fashion (including pictures)
- The patient will be able to **view the data collected** from a FitBit activity tracker in the application: the number of steps and the data about his/her sleep quality. This data is also sent to the MyPal server.
- The patient can enter his/her **medications** in the app to define reminder times for taking the medication in order to receive reminders in the defined times.
- The patient can use the MyPal **search engine** to search for content related to her/his disease using advanced search techniques for medical terms (19). The MyPal search engine has been designed to deliver accurate sources to patient that have need identified and validated by the team of the HCPs.

The app is provided in five languages: in Greek, Italian, Swedish, Czech and English (only for demonstration). The MyPal Adult app is being implemented with Flutter^1^ and uses an SQLite database.

### MyPal Web Application for the Healthcare Professionals

The MyPal web application for the healthcare professionals was developed as the central access tool of the MyPal platform for clinicians and nurses. A user centered design was adopted for the development and healthcare professionals were involved from the early stages of the design. The web application is responsive and accessible through smartphones, tables, and desktop computers and is available in five languages Greek, Italian, Swedish, Czech, and English (only for demonstration purposes). The web application provides to healthcare professionals interactive tools based on data and visual analytics, reinforcing their actions through the identification of important deviations in the patient's symptoms and quality of life. The goal of the web application for the HCP is to reinforce patient-clinician communication and improve symptom management. The potential improvement in symptom management and relief is based on meaningful interpretation of PROs which are fed directly and in real-time at the point of care. Furthermore, the exploitation of these heterogeneous data provides long-term and consistent health information along the disease timeline.

**Figure 4:**
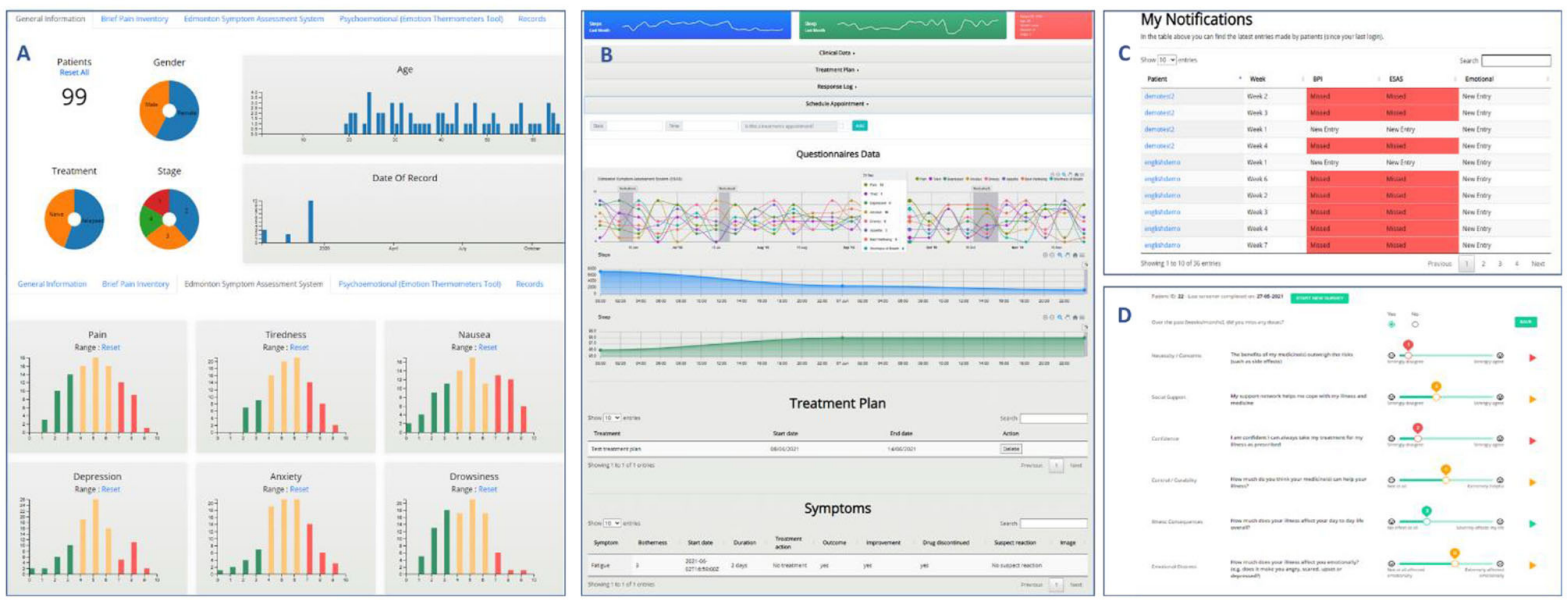
MyPal HCP web application screenshots [**(A)** the aggregated dashboard, **(B)** the individual dashboard, **(C)** notifications, **(D)** the discussion tool].

The web application for the healthcare professionals consists of various components including notifications, aggregated dashboard, individual's dashboard, discussion tool, CRF cost form and upload medical documents. Figure 4 shows some of the main tools for the HCP web application while in the following we provide a short description of each tool:

- ***Notifications*** page (Figure 4C) alerts the healthcare professional regarding the PROs that were completed or missed by the patients during each week of the study. The HCP can interact with the notifications by navigating through the available pages, sorting and searching patients or PROs.
- ***The aggregated dashboard*** page (Figure 4A) provides information for the patients of the clinical study using interactive and dynamic graphs and descriptive statistics of his/her patients. The visualized information includes demographics, treatment, stage of treatment and scores of the completed PROs and is based on a content-aware analytics framework for open health data (20). The healthcare professional is able to interact with the graphs and create subgroups of patients by choosing any of the available attributes. Finally, the patients PROs scores are presented in a table and abnormal scores are highlighted.
- ***The individual's dashboard*** (Figure 4B) presents information of each patient separately through interactive graphs and tables. Patient's PROs scores and lifestyle data (sleep, steps) are visualized as time-oriented data with a variety of interaction techniques such as zooming, grouping, downloading data etc. Moreover, the healthcare professional can insert through forms patient information, such as treatment plans, clinician appointments and clinician notes, which are also visualized as part of the graphs or tables. Finally, symptom information (bothersomeness, date, duration, images etc.) reported by the patient through the mobile application are presented.
- ***The discussion tool*** (Figure 4D) interprets and prioritizes patient responses to a Non-Adherence risk screener that assesses key patient risk factors related to non-adherence (known as the Treatment Support Survey as presented through the MyPal patient app) to help optimize patient adherence to CLL or MDS medication. During patient appointments, physicians can review the latest patient responses and also collect data about whether the patient has recently missed any doses of their CLL or MDS medication. The Discussion Tool helps the HCP to focus their conversations and deliver personalized interventions and advice on topics of highest importance at that time. HCPs click through the highest priority topics to follow step-by-step discussion guidance and provide a patient-friendly summary PDF of the advice and other local resources where available. If the patient has been non-adherent to medication, the content in the Discussion Tool is further tailored to address this challenge in more detail.
- ***The upload of medical documents*** is the tool that feeds content to the search engine for the patients and is available to the patient from the MyPal mobile app, enabling them to search for high quality, useful information. The search engine provide health related content to patients from a validated set of relevant documents using a natural language framework for medical informatics (19) and personalized recommendations (21).
- ***The CRF cost form*** is a questionnaire for the economic evaluation. It is based on the comparative measurements (and change over time) of health-related quality of life as well as costs for the intervention vs. non-intervention cohorts. The CRF cost form is completed by the clinician for a specific patient during the regular visit of the patient to the clinic.

### MyPal Backend and Security

The back-end infrastructure of MyPal consists mainly of a database to store MyPal data, an authentication server based on OAuth2.0 and software modules that are necessary to support the functionality of these tools. An audit/log mechanism is in place which records all the transactions of the users supporting the visibility and transparency, while the administrator has in his/her disposal a UI for the management of the users, overview of the audits and a testbed for all the available REST services.

**Figure 5:**
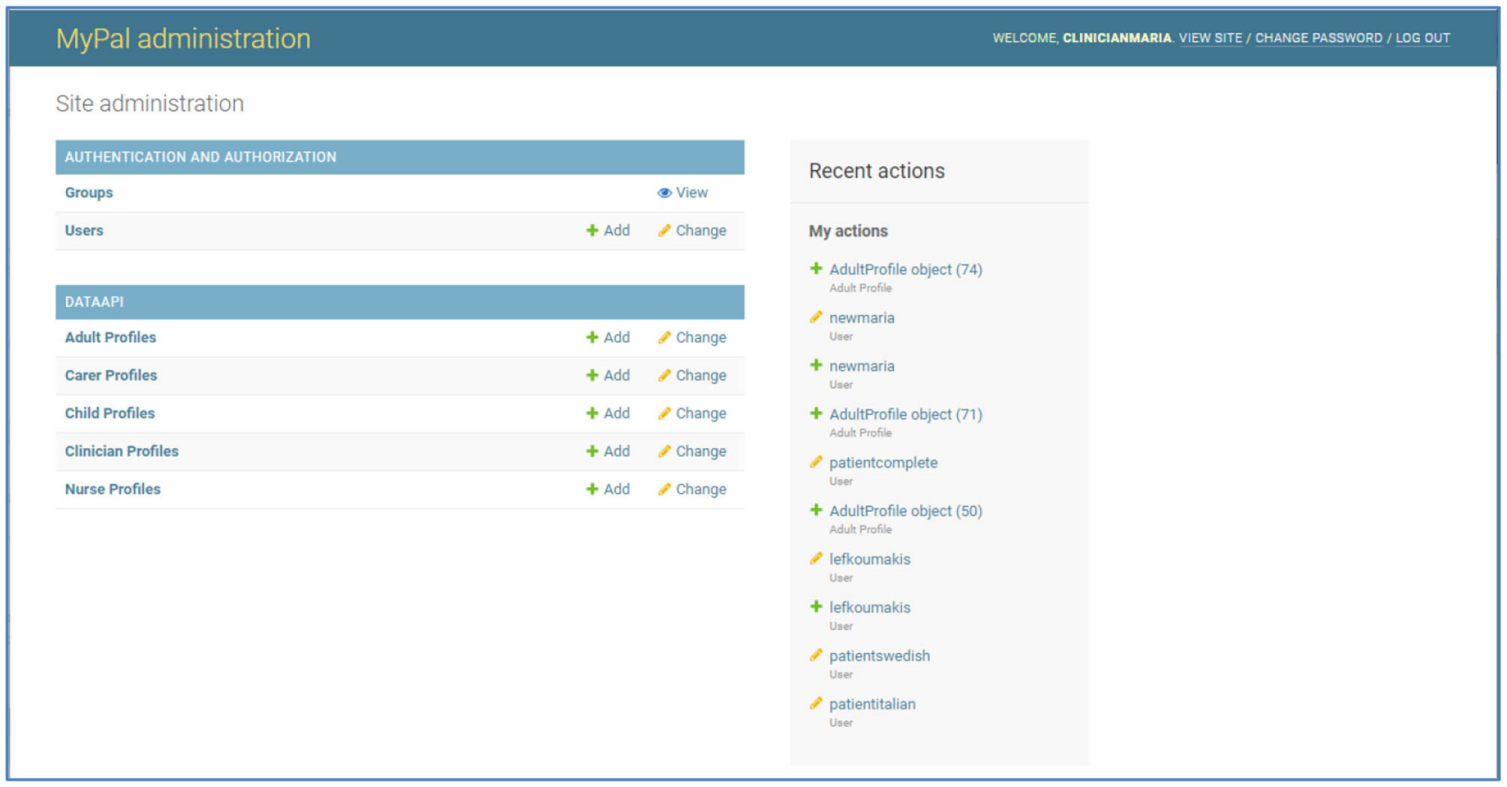
MyPal administrator panel.

At each clinical site the MyPal administrator can use the platform to add new users. In Figure 5 we can see the initial screen of the “User administration panel,” Some of these options might not be available to a clinical site depending on the MyPal studies running at this site (ADULT, CHILD) and the role/group of the user account (Administrator, Clinician, Nurse). The administrator has the following options/functionalities from the user interface of the backend:

- In the section “Groups” the administrator can see a list of group categories. Usually there is no need to view/modify anything in this section; these have been configured for MyPal during the initial installation of the platform.
- In the section “Users” the administrator can view a list of the existing users (user accounts' pseudonym and only with the minimum data) or add/modify a user account.

## Validation

For security privacy and legal reasons, it was decided that each clinical site will have an independent deployment in their premises. With local deployments in each clinical site (i.e., hospital) a separate instance of the software platform is installed in order to collect and store the data of each clinical study to a dedicated database, located at the corresponding hospital where the patients are enrolled. Scripts, manuals and virtual machines were provided to the IT teams of each hospital from the MyPal developers for the local deployments. Local deployments provide separation of datasets per clinical site and isolation of any security issues, and no risks from data transfer. Nevertheless, local/independent installations have also drawbacks such as more effort for the multiple deployments and extra processing and anonymization of the datasets for the evaluation of the results.

**Figure 6:**
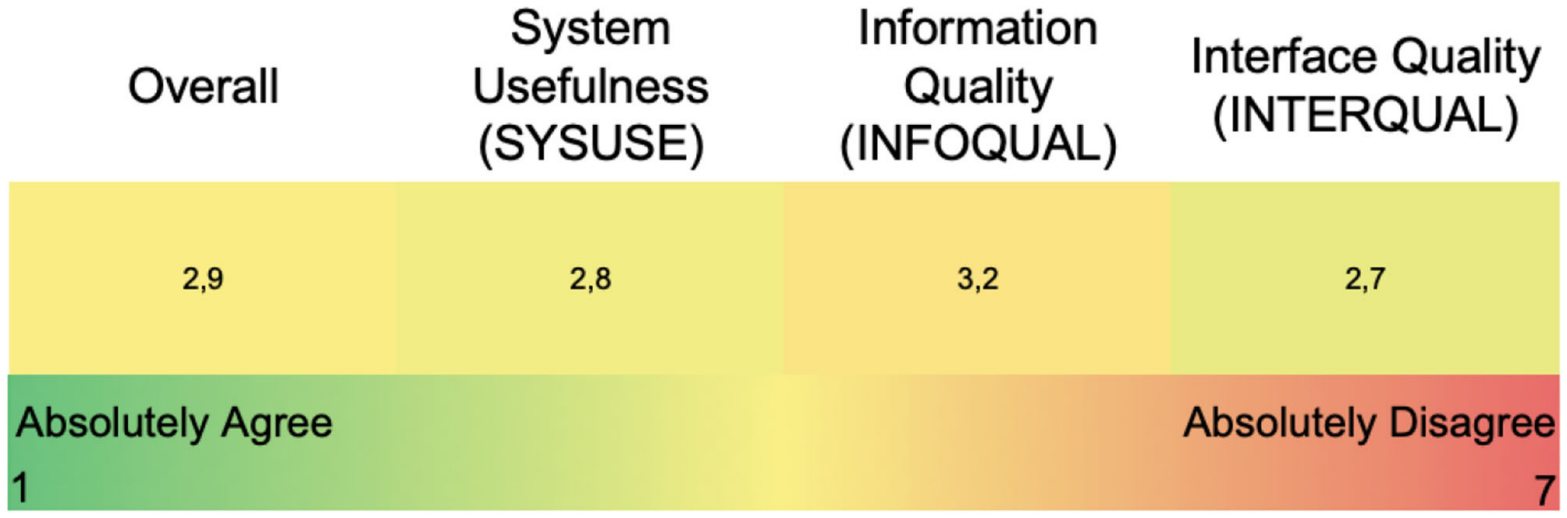
PSSUQ scores for the HCP web application (overall and sub-scores).

Regarding validation, seven virtual validation workshops with HCPs were conducted to validate the HCP web application; five of them were involved in the MyPal-Adult study and two in the MyPal-Child study. However, since HCP part of the MyPal web platform is almost identical for the two studies, the results of the validation workshops were consolidated. The vast majority of the HCPs involved, managed to complete the scenario-based actions mostly without assistance from the validation expert. The initial patient registration proved to be the most confusing, which later led to the creation of an “HCP Quick Reference Guide” to clarify the process. Moreover, the “think aloud” approach as the HCPs performed their tasks, offered several insights on the intuitiveness of the platform's design; the interactive features of the dynamic graphs presented in the aggregated and individual's dashboards were not always apparent although once familiar with them, the HCPs expressed very positive feedback for their usefulness. The need for a consistent user experience, intuitive design and adequate communication of information (color coding of symptom scales, warnings and error messages etc.) was also regularly stressed during the workshops. The PSSUQ score (22) was calculated at 2.9 overall, with the 3 sub-scales (System Usefulness, Information Quality, and Interface Quality) at 2.8, 3.2, and 2.7 respectively as seen in Figure 6.

**Figure 7:**
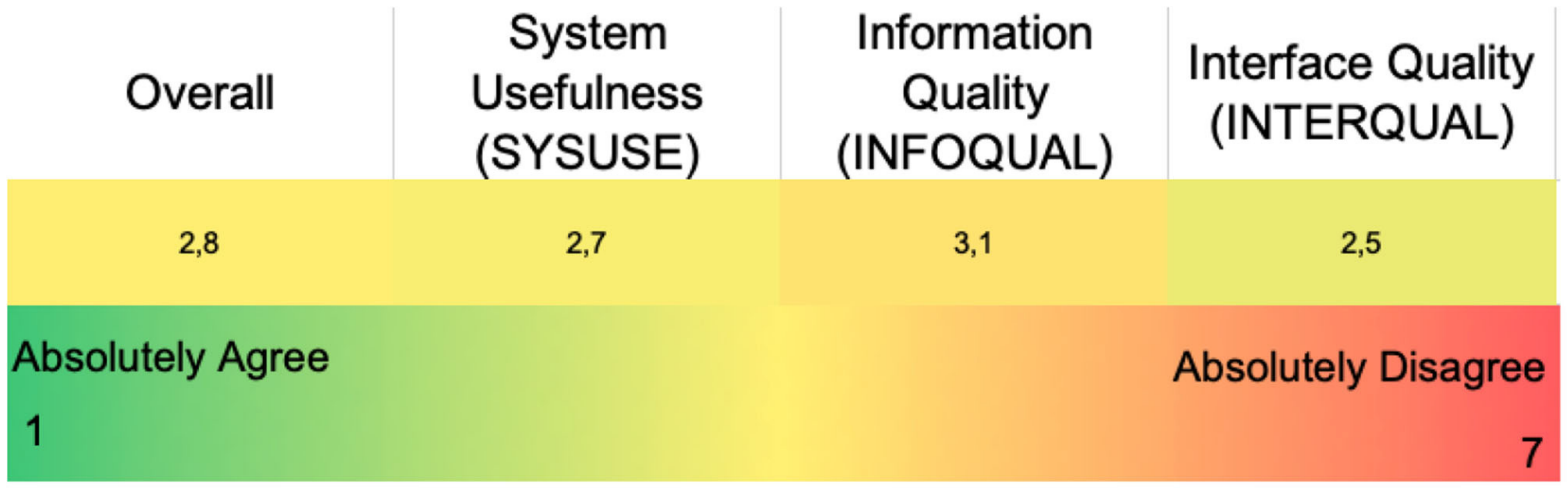
PSSUQ scores for the patient mobile app (overall and sub-scores).

Nine patients in total participated in the validation focus groups, as mentioned in the Methods section. After being presented with the validation scenario (similar to the vignettes used during the participatory design phase) the patients were asked to perform a series of actions on the MyPal mobile app. All of the participants successfully completed the actions with the majority of them requiring no assistance in doing so. As with the HCP web platform's graphs, the dynamic, color coded controls for answering the ePROs were met with enthusiasm. However, for the older patients, the rather small font size of the application, as well as some interactions/gestures–like double-tapping and scrolling through various menus—seemed to cause frustration. The spontaneous symptom report confused a lot of the patients, since they were unfamiliar with the terms mentioned in the menu (e.g., “Discontinuation,” “Rechallenged” etc.). Finally, the two quick reference guides for the MyPal mobile app exploited the feedback gathered during the validation sessions. The overall PSSUQ score was 2.8 with the subfactors being 2.7, 3.1, and 2.5 for the System Usefulness, Information Quality and Interface Quality, respectively, as shown in Figure 7.

## Discussion

After a cancer diagnosis, patients and their families face a series of challenges such as coping with fear and anxiety associated with a life-threatening disease and having to make difficult decisions about treatments (23). Communication with the healthcare team and the attending physician is very important throughout the disease trajectory, especially when decisions are to be made or transitions are taking place, for example when a new therapy option is discussed or when the goal of care changes. Physicians are an important source of information which can contribute to the development of the patient's model of disease, according to which he/she will devise a plan of action, employ coping strategies and emotionally process the experience (24): for instance, it has been suggested that patients with hematological malignancies, a particular focus of MyPal, experience difficulties in understanding health-related information, perhaps due to the fact that their disease lacks a specific focus. This coupled with the fact that many treatment strategies are characterized by uncertainty can prevent them from actively participating in the decision-making process (25).

On the other hand, healthcare providers encounter a lot of challenges in the provision of care to patients with cancer, a chronic, complex, and life-limiting disease. Communication with patients can be demanding across the disease trajectory from the moment of disclosure of cancer diagnosis, to the administration of treatment, treatment failure or relapse as well as the provision of any act of supportive palliative care throughout (26).

Considering the above, it is obvious that more sophisticated and thorough approaches are urgently needed for refined cancer management. A paradigm-shift is occurring in healthcare (27) whereby preventive, pre-emptive, and predictive healthcare decisions are increasingly taken in a pervasive, participatory and personalized manner. However, such a paradigm-shift is not possible using traditional tools and processes; it requires instead the patient's active engagement in managing his/her health. To this end, novel methodologies have been proposed to support the communication between patients and physicians. For example, communication tools can offer guidance to healthcare professionals aiming to communicate evidence-based medical information but also facilitate the exploration of patients' preferences with regards to treatment options (28). Furthermore, decision aids can not only improve patients' knowledge and reduce their anxiety levels but also promote patients' active participation in care decisions and significantly reduce decision-making conflicts with healthcare professionals (29). Finally, tools can promote earlier recognition of clinical complications such as emergency event alert systems and monitoring /reporting of drug adverse events, thus ensuring prompt and accurate patient-physician communication and improved outpatient care (30).

MyPal is one of the few research projects that proposes an eHealth intervention and evaluates it with international clinical trials across Europe. The volume and range of tasks related to the organizational and technical aspects of on-site preparations raised a number of challenges especially with the decision to support five independent deployments, one at each clinical site. Fortunately, the expertise and active involvement of all clinical and technical partners was essential to overcome these challenges.

We must acknowledge that data collection and data handling, especially for information coming from patients, pose various challenges. For that reason, MyPal applied widely accepted risk management approaches (i.e., ISO 14971) during the Data Protection Impact Assessment process.

Another challenge is the Coronavirus COVID-19 pandemic that delayed the commencement of the clinical studies. Until now, all participating hospitals are using their resources to deal with the emergency at hand and patients with serious conditions are advised against visiting the hospital unless it is absolutely necessary. Since MyPal digital intervention supports remote reporting of health-related data from patients, the clinical trials started with a small delay taking into consideration all the protocols for COVID-19.

The validation phase proved to be essential to the development cycle of the MyPal platform, continuing the participatory approach that has been adopted during the design phase. Both the HCP virtual workshops and the patient focus groups, using the MyPal solution well-before the clinical trials, offered valuable insights for the users' actual experiences. The HCP web application and the mobile app were both very favorably received by the participants and their perceived value regarding PROs was validated. Several usability issues regarding functionality and appearance aspects were revealed and elucidated into clear action points for each development team and thus resolved in a timely manner.

Currently the MyPal clinical trials in all five hospitals are running and it seems that there may be some differences between individual clinical sites in terms of recruitment rate. In some hospitals, for example, out of all the patients that meet the study criteria, only a very few are willing to participate. Patients are not familiar with mobile devices, they are used to classic phones only, which makes them ineligible for the study. All the patients above 60 years are reluctant to use modern technologies and do not seem to trust them. Younger patients, who are more accepting and capable of using mobile device, are often a part of another clinical study, or are too busy or generally not interested in being observed at higher frequency by the HCP. A high proportion of patients with CLL are in watch and wait mode so not receiving any treatment, therefore qualifying as not eligible to the study protocol. Another drawback for the recruitment is that the patients are not willing to attend the clinic every month especially with the COVID-19 pandemic. In some cases, it seems difficult to persuade patients to take part in the study, since they do not feel any need to be more involved. This passivity in terms of patients' engagement might be specific to the central Europe environment.

As a whole, we argue that the train of new clinical operational paradigms (Patient Reported Outcome Measures, Patient Reported Experience Measures, Decentralized Clinical Trials etc.) based on emerging technical paradigms (Federated Machine Learning, blockchain etc.) is already moving. However, due to the huge volatility and speed of technical developments, their actual integration as part of every-day clinical routine is far from trivial, raising cross-cutting issues going beyond technical or operational sciences silos, including aspects of both (i.e., usability and acceptability of applications, information security issues and how these are translated in patient safety risks etc.). To this end, the evaluation of eHealth interventions via clinical studies is crucial, and as such we argue that MyPal could provide a useful example both in technical and methodological terms.

## Conclusions

The diversity of cancer patient needs for palliative care is enormous. A patient-centered approach for palliative care should take into account many aspects, such as the spectrum of symptoms and clinical manifestations of the particular disease, the patient's age and overall physical condition (including comorbidities), the treatment aim (e.g., cure vs. disease control), the medication plan and its impact in QoL, life expectancy, lifestyle and exposures (including concomitant medication) as well as patient preferences, health literacy, etc. Some of the above aspects, such as the experience of adverse drug effects, are dynamic by nature, adding further difficulties in adequately addressing the individual patient needs for palliative care. MyPal aspires to provide such a patient-centered approach for palliative care of cancer patients in capturing more accurately their symptoms/conditions and communicating them in a seamless and effective way to their healthcare providers. While there are various PRO systems available, they typically lack the required flexibility to address the individual patient needs, hampering their potential due to their inherent “one-size-fits-all” approach.

MyPal focused on the thorough understanding of the varying patient needs across disease trajectories and designed a comprehensive, adaptive intervention to accommodate these needs. MyPal successfully developed and deployed a digital intervention for palliative care cancer patients overcoming several technical challenges with the continues collaboration of the clinical and technical partners of the project. Apart from the technical challenges of such an effort, one must be prepared for unpredictable parameters, such as COVID-19, and provide flexible solutions.

Through the validation activities, MyPal not only confirmed the perceived usefulness of the developed digital tools but also achieved higher levels of user acceptance by implementing modifications according to the end-user's feedback. It seems that clear communication with the end user through consistent menus, intuitive visual cues and notification messages is key to a positive user experience that ensures the engagement with the MyPal platform.

Our validation results demonstrate that such a digital platform for palliative care can improve the QoL of the patient and improve the communication between patient and physicians, a finding that is in line with literature. MyPal will evaluate a digital intervention in adults suffering from haematologic malignancies through clinical trials that are conducted in diverse healthcare settings across Europe.

## Data Availability Statement

The original contributions presented in the study are included in the article/supplementary material, further inquiries can be directed to the corresponding author/s.

## Author Contributions

LK: conceptualization, methodology, and writing—original draft. FS, MC, PA, and GZ: methodology and implementation. PB, JD, and EK: validation. MS: implementation. CKak: validation and writing. CKar: conceptualization, methodology, and validation. LS: conceptualization and validation. KM: supervision, conceptualization, and review. PN: supervision, review, and project administration. All authors contributed to the article and approved the submitted version.

## Funding

Research supported by the MyPal project which has received funding from the European Union's Horizon 2020 research and innovation program under the grant agreement No 825872.

## Author Disclaimer

This article reflects only the author's view. The Commission is not responsible for any use that may be made of the information it contains.

## Conflict of Interest

The authors declare that the research was conducted in the absence of any commercial or financial relationships that could be construed as a potential conflict of interest.

## Publisher's Note

All claims expressed in this article are solely those of the authors and do not necessarily represent those of their affiliated organizations, or those of the publisher, the editors and the reviewers. Any product that may be evaluated in this article, or claim that may be made by its manufacturer, is not guaranteed or endorsed by the publisher.

## Footnotes

^1^Available online at: https://flutter.dev/.
